# Case Report of an Infectious Aortic Aneurysm Following Intravesical Bacillus Calmette‐Guérin Therapy After Transurethral Resection of a Bladder Tumor

**DOI:** 10.1002/iju5.70176

**Published:** 2026-05-07

**Authors:** Daisuke Muto, Tsuyoshi Matsuda, Shintaro Mori, Kyohei Araki, Yuichiro Nakamura, Kensuke Mitsunari, Tomohiro Matsuo, Kojiro Ohba, Yasushi Mochizuki, Ryoichi Imamura

**Affiliations:** ^1^ Department of Urology Nagasaki University Graduate School of Biomedical Sciences Nagasaki Japan

**Keywords:** bladder cancer, infectious abdominal aortic aneurysm, intravesical BCG therapy

## Abstract

**Introduction:**

Intravesical Bacillus Calmette‐Guérin (BCG) therapy is an established treatment for non‐muscle‐invasive bladder cancer. Although systemic BCG infection is rare, infectious aortic aneurysm is a potentially fatal complication.

**Case Presentation:**

A man in his 70s underwent transurethral resection of a bladder tumor followed by intravesical BCG therapy. One year later, computed tomography revealed a saccular abdominal aortic aneurysm with extensive periaortic inflammation and an iliopsoas abscess. Polymerase chain reaction analysis confirmed *Mycobacterium bovis* BCG infection. Despite antituberculosis therapy, aneurysmal rupture with abscess progression occurred, necessitating prosthetic graft replacement with omental flap coverage. The patient recovered without recurrence after prolonged therapy.

**Conclusion:**

BCG‐related infectious aortic aneurysm may develop even in the absence of fever or marked inflammatory response. Clinicians should suspect and consider early CT evaluation in patients with a history of intravesical BCG therapy when unexplained retroperitoneal or vascular abnormalities are identified, regardless of inflammatory status.

## Keynote Message

BCG‐associated infectious aortic aneurysm may develop even in the absence of fever or marked inflammatory response; therefore, clinicians should maintain a high index of suspicion and consider early CT evaluation in patients with a history of intravesical BCG therapy when unexplained retroperitoneal or vascular abnormalities are detected.

**Abbreviations**
BCG: Bacillus Calmette‐Guérin
CT: Computed tomography
EMB: Ethambutol
INH: Isoniazid
RFP: Rifampicin
TB‐PCR: Tuberculosis polymerase chain reaction
TURBT: Transurethral resection of bladder tumor

## 1 Introduction

Intravesical Bacillus Calmette‐Guérin (BCG) instillation therapy has been used for decades as an effective adjuvant treatment for non‐muscle‐invasive bladder cancer, with well‐established efficacy in reducing tumor recurrence [1]. Although considered safe, systemic BCG infection occurs in < 1% of cases [1], and nearly 30% of these present with disseminated disease [2]. Among the disseminated manifestations, infectious aortic aneurysm is exceedingly rare but associated with a high risk of fatal rupture [3]. Because its clinical presentation is often nonspecific, diagnosis can easily be delayed. The present report describes a case of an infectious abdominal aortic aneurysm that developed after intravesical BCG instillation therapy and highlights the critical importance of early recognition, accurate diagnosis, and timely therapeutic intervention.

## 2 Case Presentation

A man in his 70s who had undergone transurethral resection of a bladder tumor (TURBT) followed by intravesical BCG instillation therapy was referred to our department one year later, after postoperative follow‐up computed tomography (CT) for congenital biliary dilation revealed a soft‐tissue mass surrounding the abdominal aorta and left iliopsoas muscle at the level of the renal arteries.

**FIGURE 1:**
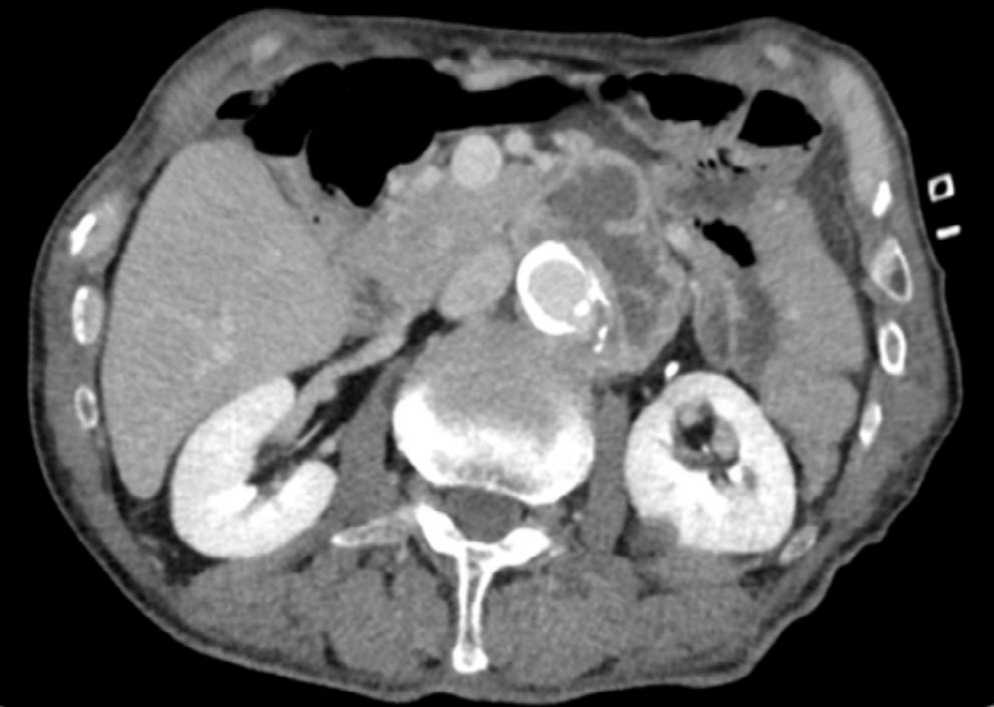
Contrast‐enhanced computed tomography at admission. Axial view showing a saccular infrarenal abdominal aortic aneurysm with marked periaortic inflammation and left iliopsoas abscess.

At admission, the patient's vital signs were stable, with blood pressure 105/80 mmHg, pulse 78/min, body temperature 36.4°C, and peripheral capillary oxygen saturation (SpO₂) of 97% on room air. Laboratory tests showed a white blood cell count of 5.1 × 10^3^/μL, C‐reactive protein of 0.97 mg/dL, and serum creatinine of 0.98 mg/dL. Contrast‐enhanced CT revealed a saccular abdominal aortic aneurysm with surrounding soft‐tissue inflammation and an associated iliopsoas abscess (Figure 1). Laparoscopic biopsy of the periaortic lesion yielded turbid intralesional fluid, and *Mycobacterium bovis* BCG was detected using tuberculosis polymerase chain reaction (TB‐PCR). Blood and mycobacterial culture results were negative.

**FIGURE 2:**
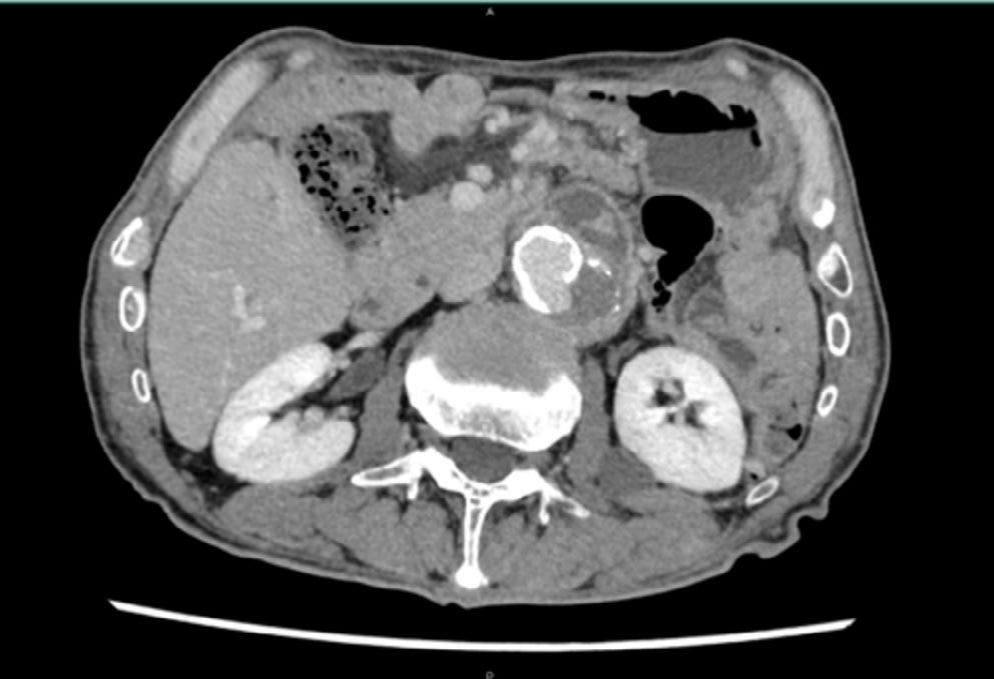
Contrast‐enhanced computed tomography at the time of aneurysmal rupture. Progressive enlargement of the abdominal aortic aneurysm and extensive spread of the surrounding abscess are observed.

**FIGURE 3:**
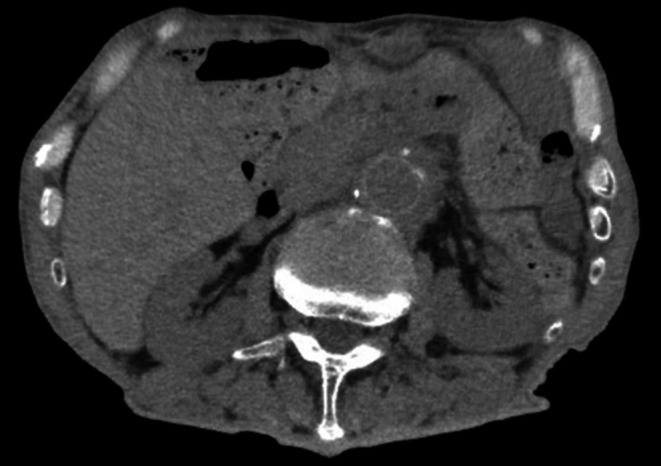
Postoperative computed tomography. Follow‐up CT 6 months after prosthetic graft replacement demonstrates complete resolution of the periaortic inflammation and no evidence of aneurysmal recurrence.

Antituberculosis therapy with isoniazid (INH), rifampicin (RFP), and ethambutol (EMB) was initiated; however, follow‐up CT at five months revealed an aneurysmal rupture with abscess progression. Given the worsening of the infection, prosthetic graft replacement of the abdominal aorta with additional omental flap coverage was performed (Figure 2). Postoperatively, the patient received intravenous INH and levofloxacin followed by a transition to oral therapy. Follow‐up blood tests and CT scans at one and six months postoperatively demonstrated resolution of inflammation and no recurrence of the aneurysm (Figure 3).

A brief timeline of the clinical course is summarized as follows:

Month 0: Completion of intravesical BCG therapy

Month 12: Incidental CT detection of periaortic mass

Month 12: TB‐PCR confirmed *Mycobacterium bovis* infection

Month 12: Initiation of antituberculosis therapy

Month 17: Aneurysmal rupture with abscess progression

Month 17: Prosthetic graft replacement with omental flap coverage

## 3 Discussion

BCG‐related infectious aortic aneurysm represents one of the rarest yet most severe complications of intravesical BCG instillation therapy. Although BCG is intended to exert its therapeutic effect locally within the bladder, mucosal disruption caused by TURBT may facilitate hematogenous dissemination [1]. Importantly, this process may occur insidiously and remain clinically silent for a prolonged period, resulting in delayed presentation without systemic symptoms such as fever or marked inflammatory response. Once disseminated, BCG can lodge in the aortic wall, inducing granulomatous inflammation that progressively weakens the vascular structure and eventually leads to aneurysm formation.

Previous reports have shown that approximately 70% of BCG‐related infectious aortic aneurysms occur in the infrarenal abdominal aorta, and approximately 40% are accompanied by iliopsoas or retroperitoneal abscesses [4]. The median onset is approximately 15 months after BCG therapy [3], and this delayed presentation constitutes a major reason for diagnostic delay. Clinical manifestations of BCG‐associated infectious aortic aneurysm are often nonspecific. While some patients present with fever or back pain, a substantial proportion may remain afebrile and exhibit only minimal inflammatory response. Therefore, reliance on fever alone as a trigger for diagnostic imaging may delay recognition of this potentially life‐threatening complication.

Contrast‐enhanced CT is essential for diagnosis as it delineates saccular aneurysm formation, periaortic inflammation, and abscess formation. However, blood cultures are frequently negative, and definitive diagnosis often requires PCR analysis of tissue specimens [5]. Negative culture results may be attributable to a low bacterial burden or prior antimicrobial exposure, which can reduce the sensitivity of conventional microbiological methods. In the present case, TB‐PCR confirmed BCG infection, and the combination of a saccular mass in the abdominal aorta and a clear history of BCG instillation therapy enabled prompt diagnosis and appropriate treatment planning.

The mainstay of treatment includes prolonged antituberculosis therapy; however, surgical intervention is mandatory in cases of structural compromise of the aortic wall or progressive infection. The initial therapy typically consists of INH, RFP, and EMB, followed by 9–12 months of maintenance therapy [6]. Surgical options include prosthetic graft replacement, extra‐anatomical bypass, autologous vein grafting, and endovascular aneurysm repair, selected according to the clinical scenario [7, 8]. In the present case, surgical intervention was ultimately required due to aneurysmal rupture and progressive abscess formation despite appropriate antituberculosis therapy, suggesting structural compromise of the aortic wall and failure of conservative management. Intraoperatively, extensive debridement of the infected periaortic tissue was performed prior to graft placement to minimize the risk of persistent infection, and omental flap coverage was used to enhance local infection control. This case underscores that BCG‐related infectious aortic aneurysms may progress even in the absence of significant inflammatory markers. PCR plays an essential role in confirming the diagnosis, and a timely transition to surgical intervention is critical for achieving favorable outcomes. In our case, contrast‐enhanced CT initially demonstrated a saccular aneurysm with surrounding inflammation (Figure 1), which progressed despite medical therapy (Figure 2), ultimately resolving after surgical intervention (Figure 3). Recent reports indicate that up to 30% of BCG‐associated infectious aneurysms may present without aneurysm‐related symptoms or fever [3, 4]. Therefore, reliance on fever alone as a trigger for imaging may delay diagnosis. In patients with a history of intravesical BCG therapy, unexplained retroperitoneal abnormalities or subtle imaging findings should prompt consideration of infectious aneurysm regardless of inflammatory status.

## 4 Conclusion

BCG‐related infectious aortic aneurysm is a rare but potentially fatal complication of intravesical BCG instillation therapy that may develop even in the absence of fever or marked inflammatory response. Early CT evaluation should be considered in patients with a history of intravesical BCG therapy when unexplained retroperitoneal or vascular abnormalities are identified. Early detection and timely therapeutic intervention are crucial for preventing catastrophic outcomes.

## Consent

We obtained informed consent from the patient.

## Conflicts of Interest

The authors declare no conflicts of interest.

## Data Availability

The data that support the findings of this study are available from the corresponding author upon reasonable request.
